# Supplementary material for: Hybrid Models and Biological Model Reduction with PyDSTool
Source: PLoS Comput Biol. 2012 Aug 9;8(8):e1002628. doi: 10.1371/journal.pcbi.1002628 (PMC3415397; doi:10.1371/journal.pcbi.1002628)
Supplement: Text S4 — Complete source code for the PyDSTool package (version 0.88.120504). Includes API documentation and help files linking to web pages. This file is identical to the current public release on Sourceforge.net. (ZIP) [file pcbi.1002628.s004.zip › PyDSTool/html/PyDSTool.Toolbox.optimizers.helpers.finite_difference.FiniteDifferencesCache-class.html]

xml version="1.0" encoding="ascii"?


PyDSTool.Toolbox.optimizers.helpers.finite\_difference.FiniteDifferencesCache


| Home | Trees | Indices | Help | PyDSTool |
| --- | --- | --- | --- | --- |

Package PyDSTool ::
Package Toolbox ::
Package optimizers ::
Package helpers ::
Module finite\_difference ::
Class FiniteDifferencesCache

[hide private]

[frames] | no frames]

# Class FiniteDifferencesCache

source code

```
               object --+    
                        |    
FiniteDifferencesFunction --+
                            |
                           FiniteDifferencesCache
```

Known Subclasses:
:   - ForwardFiniteDifferencesCache

---

General class for recognition by ParamEst as a function with a
non-explicit derivative. Uses a cache to save recomputation of most
recent values.


|  |  |
| --- | --- |
| Instance Methods | [hide private] |

|  |  |
| --- | --- |
| \_\_call\_\_(self, params) | source code |

|  |  |
| --- | --- |
| \_\_init\_\_(self, eps=1e-07, \*args, \*\*kwargs)  Creates the function :... | source code |

|  |  |
| --- | --- |
| residual(self, p, extra\_args=None) | source code |

|  |  |
| --- | --- |
| \_res\_fn(self, p, extra\_args=None) | source code |

**Inherited from `object`**:
`__delattr__`,
`__getattribute__`,
`__hash__`,
`__new__`,
`__reduce__`,
`__reduce_ex__`,
`__repr__`,
`__setattr__`,
`__str__`


|  |  |
| --- | --- |
| Properties | [hide private] |

**Inherited from `object`**:
`__class__`


|  |  |
| --- | --- |
| Method Details | [hide private] |

|  |  |
| --- | --- |
| \_\_init\_\_(self, eps=1e-07, \*args, \*\*kwargs)  *(Constructor)* | source code |

```
Creates the function :
- eps is the scale at which the function varies by O(1) in each
parameter direction
- grad_ratio_tol (optional, default = 10) is the relative change in any direction
after which the function is deemed to have changed non-smoothly, so that gradient in
that direction will be ignored this step
```

Overrides: object.\_\_init\_\_

|  |  |
| --- | --- |
| residual(self, p, extra\_args=None) | source code |

Overrides: FiniteDifferencesFunction.residual

  


| Home | Trees | Indices | Help | PyDSTool |
| --- | --- | --- | --- | --- |

|  |  |
| --- | --- |
| Generated by Epydoc 3.0.1 on Fri May 4 15:24:09 2012 | http://epydoc.sourceforge.net |
